# Using Life Story Techniques with Diverse Communities: Lessons Learned from a Focus Group Study

**DOI:** 10.1093/geroni/igab046.3068

**Published:** 2021-12-17

**Authors:** Christa Wilk, Ashlee Cordell, Silvia Orsulic-Jeras, Sara Powers, Farida Ejaz, Lisbeth Sanders

**Affiliations:** 1 Benjamin Rose Institute, Cleveland, Ohio, United States; 2 Benjamin Rose Institute on Aging, Cleveland, Ohio, United States; 3 LifeBio, Marysville, Ohio, United States

## Abstract

Providing high quality, cost-effective dementia care remains a major health challenge. Life story work, used in residential care settings, helps engage persons living with dementia (PWD) at a low cost with minimal staff burden. LifeBio, one such intervention, is designed to elicit life history data and care preference information through comprehensive life story interviews. LifeBio Memory, an adaptation of LifeBio, utilizes novel speech-to-text technology to process life story data more efficiently. Seven focus groups were conducted to evaluate the acceptability and feasibility of LifeBio Memory. Three types of focus groups were held (n=35) and audio recorded: 1) One group of early-stage PWDs (n=5); 2) Two groups of current and former users of original LifeBio (n = 12); and 3) Four groups of residential care staff and directors (n=18). Sessions were transcribed and thematic analyses were conducted. Findings indicated high levels of acceptability and feasibility of LifeBio Memory. Further, a secondary theme emerged signaling the need to prioritize the emotional safety of PWDs participating in life story work. This poster will discuss: 1) the life story interview process, 2) identifying PWDs who would most benefit from a life story program, 3) involving family and staff care partners to identify sensitive topics, 4) interview question design, selection and order, and 5) expanding life story work across diverse communities. Discussion will highlight the importance of protecting the emotional well-being of marginalized communities by identifying potential underlying traumas that could impact the safe delivery of otherwise effective life story interventions.

